# Diabetic Foot and Fungal Infections: Etiology and Management from a Dermatologic Perspective

**DOI:** 10.3390/jof10080577

**Published:** 2024-08-15

**Authors:** Aditya K. Gupta, Avner Shemer, Vasiliki Economopoulos, Mesbah Talukder

**Affiliations:** 1 Division of Dermatology, Department of Medicine, Temerty Faculty of Medicine, University of Toronto, Toronto, ON M5S 3H2, Canada; 2 Mediprobe Research Inc., London, ON N5X 2P1, Canada; veconomopoulos@mediproberesearch.com (V.E.); mtalukder@mediproberesearch.com (M.T.); 3 Department of Dermatology, Sheba Medical Center, Tel-Hashomer, Ramat-Gan 52621, Israel; ashemer1@gmail.com; 4 Sackler Faculty of Medicine, Tel Aviv University, Tel Aviv 6997801, Israel; 5 Department of Medical Biophysics, Schulich School of Medicine and Dentistry, Western University, London, ON N6A 3K7, Canada; 6 School of Pharmacy, BRAC University, Dhaka 1212, Bangladesh

**Keywords:** diabetes mellitus, onychomycosis, tinea pedis, diabetic foot infection, diabetic foot ulcer

## Abstract

Diabetes Mellitus (DM) is a significant global concern. Many diabetic patients will experience complications due to angiopathy, neuropathy, and immune dysfunction, namely diabetic foot ulcers (DFU) and diabetic foot infections (DFI), which can result in lower limb amputation and potentially death. The prevalence of common superficial fungal infections, such as tinea pedis and onychomycosis, can directly increase a diabetic patient’s risk of developing both DFU and DFI. In this review article, we discuss the etiology of diabetic foot complications as well as considerations for both screening and management. We also discuss the role of the dermatologist within a multidisciplinary care team in prescribing and managing treatments for tinea pedis and onychomycosis infections within this patient population. We believe that reducing the burden of these fungal infections in the context of the diabetic foot will help reduce DFU and DFI complications and their associated morbidity and mortality.

## 1. Introduction

**Figure 1:**
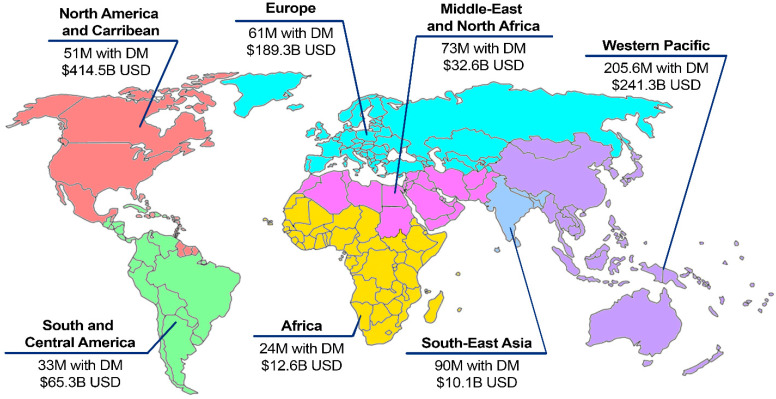
Impact of Diabetes Mellitus globally. Constructed using IDF Atlas 2021 data [1], illustrating the number of patients with DM in each region and healthcare expenditure.

Diabetes Mellitus (DM) represents a significant global health concern, with approximately 537 million adults globally living with DM in 2021, with an associated cost of approximately USD $966 billion [1,2]. In 2021, there were 38.1 million adults over the age of 18 in the United States living with DM [3], which has significant implications for the healthcare system, particularly diabetic foot complications, which lead to high healthcare costs and resource utilization [4]. Additionally, DM has varying impacts on patients from different socioeconomic backgrounds [5,6,7]. In the US, the rate of DM is highest within indigenous populations, followed by Hispanic and African American populations, with the lowest rates seen in White and Asian populations [5,6,7]. This disparity is also apparent in the health outcomes that these patients face [7], particularly as health insurance coverage within the US varies with ethnicity, income, and education level [8]. These disparities lead to an increased prevalence of diabetic foot complications in these disadvantaged populations [6,9,10,11,12]. Figure 1 illustrates the effects of diabetes globally.

The hyperglycemic state that is characteristic of both type 1 and type 2 DM leads to changes affecting many organs, including but not limited to the eyes, kidneys, skin, peripheral nervous system, and cardiovascular system, potentially resulting in peripheral arterial disease (PAD), diabetic peripheral neuropathy (DPN), skin abnormalities, and immune dysfunction [10,13,14,15].

One of the major complications patients with poorly managed long-term DM face is developing a diabetic foot ulcer (DFU). These ulcers lead to significant morbidity and mortality. Subjects with DFU experience high rates of infection that are thought to precede approximately 85% of lower limb amputations [14], with many resulting in death [6]. Between 19% and 34% of diabetics will develop a DFU during their lives, which is up to 26.1 million patients globally [7]. Up to 20% of patients with a DFU will require lower limb amputation [6], and up to 80% of these patients will die within 5 years of amputation [16]. PAD is a major risk factor for amputation and mortality.

DFUs develop due to several factors, including PAD, DPN and associated joint and foot abnormalities, skin abnormalities and damage, impaired wound healing, and diabetic foot infection (DFI) [10,15]. DFI is an infection of soft tissue or bone distal to the malleoli in a diabetic patient [17] and can be the result of, rather than the cause of, a DFU [17]. Seemingly minor infections for non-diabetic patients, such as onychomycosis and tinea pedis, can have a significant impact on diabetics [14]. In the diabetic patient, the skin’s normal barrier and immune function, as well as wound healing capacity, are compromised [15,18]. In diabetic patients, the most common complication requiring hospitalization is a DFI, with diabetic foot osteomyelitis (DFO) being present in 44-68% of the hospitalizations [17].

In this review, we will discuss the etiology of diabetes-induced physical changes generally, as well as those specific to the skin and their associated foot complications. We will also discuss the role of the dermatologist in managing patients with diabetes-associated foot abnormalities and preventing future complications, including DFUs, particularly in patients with limited access to care.

## 2. Etiology/Pathobiology of Diabetic Foot Complications

The changes that occur in DM are precipitated by hyperglycemia, with poor long-term control increasing the risk for debilitating complications [17,19,20,21]. The hyperglycemic state affects the vasculature, peripheral nervous system, and immune function, which in turn affects many organ systems, including—but not limited to—the brain, heart, kidneys, eyes, and skin [13,15,17,19,20,21]. These changes eventually lead to significant pathologies within the lower limbs that lead to severe complications if not managed [10,15,18]. Following, we discuss the diabetes-associated changes that occur and their impact on the skin.

### 2.1. Vascular Function

#### 2.1.1. Development of Vascular Disease

Vascular disease is a common occurrence in DM, with 10% to 20% of diabetics experiencing symptomatic PAD. However, many patients with vascular disease are asymptomatic, possibly underestimating the true prevalence rate [10,22]. Patients can experience both macro- and microvascular disease, leading to atherosclerosis and ischemia [23]. Inflammation due to hyperglycemia, dyslipidemia, hypertension, and insulin resistance contributes to the development of vascular disease [10,24]. Hyperglycemia causes injury to endothelial cells, which generates reactive oxygen species (ROS) and advanced glycation end-products (AGE). ROS and AGE lead to vascular and systemic inflammation, which contributes further to vascular damage and the development of atherosclerosis [10,19,23,25].

#### 2.1.2. Macrovascular Changes

Macrovascular complications range from PAD (which is our main focus), stroke, coronary artery disease, and heart failure; these have significant impacts on patient morbidity and mortality in DM [23,26,27,28].

PAD in DM frequently presents as partial or full occlusion of peripheral arteries due to atherosclerosis, with vessels below the knee affected most often [20,26,29]. The reduction in perfusion seen in diabetic PAD reduces nutrient supply and immune access while increasing hypoxia. In the extremities, this can contribute to poor infection control, potential ulcer formation, and gangrene, as well as possible amputation and death [6,18].

#### 2.1.3. Microvascular Changes

Hyperglycemia and dyslipidemia that occur in diabetic patients lead to microvascular injury, specifically affecting intraluminal endothelial cells through mechanisms listed above in Section 2.1.1 [24,30]. This results in vascular inflammation and vasoconstriction, which decreases perfusion at the level of the capillary bed [30,31]. There is thickening of the basal membrane in the capillary bed, resulting in hypoxia and reduced nutrient exchange, predisposing to DFU formation [17,25,31,32].

### 2.2. Neurological Function

#### 2.2.1. Development of Neuropathies

Neuropathy is a common consequence of DM, particularly with long-term uncontrolled hyperglycemia [10,13,33]. Additionally, factors such as uncontrolled hypertension and hyperlipidemia may also play a role in the development of neuropathies by increasing systemic inflammation [13,27,34]. Hyperglycemia within peripheral neurons leads to ROS and AGE formation, causing neuronal inflammation and altered cellular function [33,35,36,37,38].

Dyslipidemia, frequently present in type 2 DM, also leads to damage within neurons by promoting systemic inflammation [27,34]. The microvasculature may contribute to neuropathy through local ischemia [37]. Ultimately, these processes lead to oxidative stress and inflammation, causing cell damage [39]. The neuronal damage that occurs can create either sensory, motor, or autonomic changes, which can have serious implications for patients, particularly in the development of DFU [10,13,17,19,33,35,37,38,40].

#### 2.2.2. Sensory Changes

In sensory neurons, insulin signaling plays a significant role in cell survival and maintenance through the activation of neurotrophic pathways [37,41]. Deficits in insulin signaling, due to a lack of insulin in type 1 and insulin resistance in type 2 DM, can lead to a drop in neurotrophic pathway activation and neuronal deterioration [37,41].

Once sensory neurons become impaired, they experience neurodegeneration and atrophy as a “dying back” pattern [37], manifesting in a stocking-like distribution [13]. Damage can present as a “numb” or “asleep” sensation, but in up to one-third of patients, it may also present as burning, tingling, or stabbing sensations [13]. In many cases, this loss of sensory function leads to a loss of pain, proprioception, and temperature perception, potentially predisposing to trauma and the occurrence of DFU [6,17,19,20].

#### 2.2.3. Motor Changes

Motor neuron impairment is typically seen in advanced cases of diabetic neuropathy, presenting as muscle weakness and atrophy in the extremities [42]. The same mechanisms discussed above in Section 2.2.2 may also play a role in the degeneration of motor neurons [41]. Degradation of motor neurons, particularly in the lower extremities and feet, causes muscle atrophy, resulting in altered biomechanics within the foot. When combined with other complications, the patient may experience motor and associated sensory deficits, such as asteatosis, with a higher probability of developing DFU [6,19,20,21,42].

#### 2.2.4. Autonomic Changes

Autonomic neuropathy can manifest as complications within the cardiovascular and sudomotor systems [33,34]. Dysfunction in the autonomic nervous system (ANS) also affects the microcirculation with a loss of sympathetic tone, creating a cycle where vascular dysfunction leads to further neurological dysfunction and vice versa [43,44]. This cycle can have significant implications, resulting in the deterioration of skin integrity.

Patients who develop sudomotor dysfunction affecting the skin may experience anhidrosis and asteatosis [13,45]. These complications can lead to the development of cracks or deep fissures, thereby creating a portal for infection [18,21].

### 2.3. Immune Dysfunction

#### 2.3.1. Inflammation in Diabetes

Chronic low-level inflammation is associated with insulin resistance and, consequently, type 2 DM [15,46,47,48]. This inflammation leads to cellular signaling and cytokine production that blunts insulin function while at the same time activating transcription and expression of additional inflammatory proteins, furthering the inflammatory cycle [46,47]. This inflammatory state contributes to both vascular dysfunction and neuropathy [13,27,34]. Additionally, hyperglycemia and insulin resistance create dysfunction within the immune system itself, impacting the patient’s ability to fight infection [15,20,46].

#### 2.3.2. Changes in Immunity

Several immune cell populations are impaired in diabetic patients in the innate immune system [15], as well as in the adaptive immune system [46]. Insulin resistance and hyperglycemia directly impact immune function, attenuating the activation and function of macrophages, neutrophils, and NK cells [48,49]. Hyperglycemia also impairs immunoglobulin function through non-enzymatic glycation [48], as well as leukocyte infiltration into tissues [46].

#### 2.3.3. Changes in Wound Healing

Wound healing is delayed in approximately 20% of DM patients [47]. The impairments present within the innate immune system directly impact wound healing [15,18]. During healing, there are disruptions in the ability of the innate immune response to switch from a pro-inflammatory, pathogen-killing state to an anti-inflammatory, regeneration-promoting state [18,29,50]. This leads to impairment of keratinocyte and fibroblast functioning, which can significantly delay healing and allow infections more time to take hold [18,29,40].

### 2.4. Overall Changes Observed in Diabetic Skin and Impact on Diabetic Foot Complications

DM-associated skin changes occur in 30% to 70% of patients [15,40]. The combination and interplay of vascular deficits, neuropathy affecting the skin, and immune dysfunction creates dry skin on the foot that is prone to cracks, fissures, callous formation, and a loss of protective sensation; these skin changes and associated infections may be the first signs of undiagnosed DM [40].

## 3. Skin Infections in the Diabetic Foot

The changes evident in the foot and skin of diabetic patients cause greater susceptibility to infection and downstream complications, affecting up to a third of patients [51]. Infections that normally would be of little concern to those without DM can have grave consequences for diabetic patients [52]. DFI can range from onychomycosis and tinea pedis to cellulitis, severe necrotizing fasciitis, and life-threatening infections [6,17,19,20,53]. It has been estimated that the sources of DFIs arise as follows: interdigital spaces (60%), secondary to onychomycosis (30%), and trauma-related (10%) [17].

### 3.1. Onychomycosis

Toenail onychomycosis is thought to occur in 22–30% of diabetic patients [52,53,54,55,56,57]. Mild onychomycosis is thought to carry a minor risk of complications; however, more severe infections are of great concern [53]. Onychomycosis can lead to thick, sharp, and brittle nails that can cause injury to the foot [14]. Additionally, erosion of the nail unit due to poorly fitting footwear can occur, particularly in the presence of sensory loss, potentially leading to ulceration and secondary infections that may involve deeper tissues and bone [53].

### 3.2. Tinea Pedis

Tinea pedis can present a significant risk to diabetic patients, where tinea pedis occurs 2.5 to 2.8 times more frequently than in the general population [14,58]. Patients tend to present with interdigital infections [59], which can lead to the development of fissures, increasing their susceptibility to secondary infections [59]. Tinea pedis is also present in up to two-thirds of DM patients with onychomycosis, further complicating the infection and treatment [14,53].

### 3.3. DF Infections

**Figure 2:**
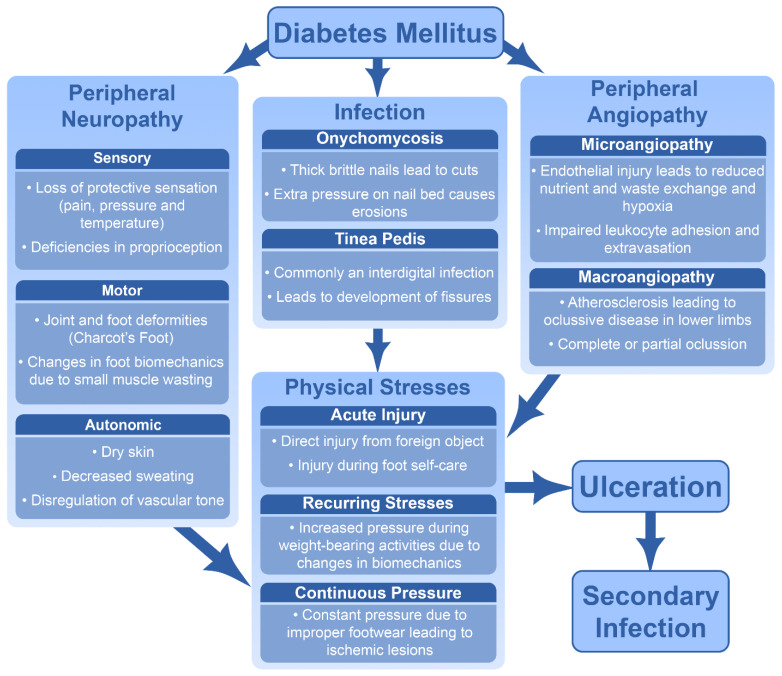
Schematic depicting the factors and pathways affecting the development of complications in DM.

Both onychomycosis and tinea pedis can lead to additional complications [58,59,60,61]. As described above in Section 3.1 and Section 3.2, these infections leave patients prone to breaks within the skin, compromising the skin’s natural barrier function [40,62,63] and eventually leading to DFUs and secondary infections [58,61,63]. These secondary infections, combined with the diabetic foot complications detailed in Section 2, can lead to serious complications. These include not only non-healing DFUs but also deep tissue and bone infections leading to lower limb amputation, as well as sepsis and death [14,64]. Figure 2 summarizes how different aspects of the diabetic foot and infection can lead to ulceration and secondary infections.

## 4. Screening for Risk of Diabetic Foot Ulcers

In diabetic patients, a comprehensive foot examination should be performed by a family physician, dermatologist, podiatrist, or other specialist. Assessing for evidence of peripheral arterial disease and neuropathy is one of the principle priorities during examination [2,19]. The physical examination should look for evidence of callouses, dry skin, thickened toe nails, abnormal hair distribution, and both interdigital and plantaris tinea pedis. The feet should also be evaluated for deformities, such as equinus deformity by evaluating dorsiflexion and plantarflexion, Charcot’s arthropathy, as well as digital deformities such as claw toes or hammer toes [2,19].

Neurological function in both extremities needs to be assessed. Specifically, loss of protective sensation (large fiber neuropathy) can be evaluated using the Semmes-Weinstein 5.07 monofilament test (10 g force) to test sensation and a 128 Hz tuning fork to test vibration perception [2,19,65,66]. When the monofilament and tuning fork tests are not possible, the Ipswich touch test can be used to assess the perception of light touch [2,65].

The vascular supply to each lower limb must be assessed. Pulses should be palpated at the posterior tibial and dorsalis pedis arteries; however, PAD cannot be ruled out if pulses are palpable. Additionally, the ankle-brachial index (ABI) should be determined for each lower limb through Doppler-recorded pressures in both of these arteries and compared to the brachial pressures [2,19,21,29,65,67,68]. If the ABI is <0.9, then PAD is strongly suspected. Calcification within these arteries, however, can create a falsely elevated ABI, and in these cases, the toe-brachial index should be used with pressures measured from the great toe. If the toe-brachial index is <0.7, then PAD is strongly suspected. PAD can also be assessed by measuring the transcutaneous oxygen pressure and skin perfusion pressure [2,21,29,65,67,68].

More specialized assessment techniques, such as the Society for Vascular Surgery’s Wound, Ischemia, Foot Infection (WIFI) classification system [6,69,70], which assesses the risk of amputation and benefit of revascularization interventions in patients with foot ulcers, are beyond the scope of this article [2,71]. Other reported classification systems include SINBAD (Site, Ischemia, Neuropathy, Bacterial Infection, and Depth), PEDIS (Perfusion (ischemia), Extent (area), Depth, Infection, Sensation (neuropathy)), Meggit-Wagner and University of Texas Systems [10,17,38,72] and we refer the reader to the appropriate literature regarding these classification systems.

## 5. General Management of the Diabetic Foot

**Figure 3:**
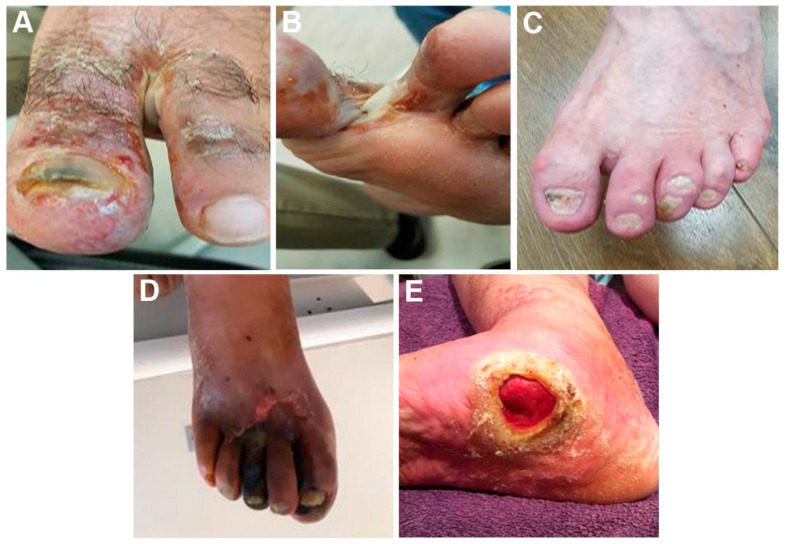
Clinical presentations of diabetic foot infections and complications. Panel (**A**)—60 years old male patient with onychomycosis (*Trichophyton rubrum*), tinea pedis complicated with secondary bacterial infection with *Staphylococcus aureus* and *Pseudomonas aeruginosa*, panel (**B**)—Same patient in panel (**A**) with tinea pedis interdigitalis contaminated also with *Candida albicans*, panel (**C**)—75 years old male patient with painless ulcer due to inappropriate shoes, panel (**D**)—40 years old male patient with unstable diabetes with severe atherosclerosis and the onset of necrosis, and panel (**E**)—60 years old male patient with painless deep diabetic ulcer where the bone can be visualized.

The major complication diabetic patients face is the development of foot ulcers and their subsequent risk for lower limb amputation. Early recognition of developing ulcers, infections, and at-risk patients is essential for reducing amputation risk and related morbidity [2,7,73]. Figure 3 depicts some clinical presentations of these diabetic foot complications. Patients will require advanced wound care, which can include debridement, specialized dressings, and wound off-loading [2,9,17,29]. Early assessment by a multidisciplinary team consisting of primary care physicians, diabetes specialists, dermatologists, podiatrists, infectious disease specialists, and surgeons in the acute phase of diabetic foot infections and ulcers increases the likelihood of positive outcomes for these patients [7,9,71,73,74].

The main goal of ulcer management is to spare the affected limb and reduce morbidity and mortality. For DFUs to heal, there needs to be adequate arterial supply, infection control, and offloading at the DFU and its vicinity [6,17,29]. In the clinical setting, this would include optimizing glycemic control, treating any active infections with oral/intravenous antibiotics and antifungal agents, surgical debridement of the wound, and addressing lower limb ischemia in patients with PAD [2,9,19,71]. Additionally, treating physicians need to be cognizant of deeper soft tissue infections and osteomyelitis, as these potentially limb-threatening infections require early and specialized treatment for optimal outcomes [17,71,75].

Revascularization therapy in patients with lower limb-threatening ischemia can increase arterial circulation to the affected limb to support wound healing. These therapies can include open vascular bypass surgery or endovascular surgery [2,18,19,20,71].

Patients with active ulcers also require specialized wound care, which can include offloading devices such as total contact casts or prefabricated knee-high walkers [2,10,19,29]. Offloading devices are still important even when ulcers are healed, but also when biomechanical changes in the foot lead to high pressure areas in patients without previous DFU. Customized footwear and orthotic inserts can help accommodate these changes and prevent future ulcerations in high-risk patients [7,9,76].

Patients with DFUs require frequent, long-term management and monitoring by a multidisciplinary care team, as healing can take anywhere from 12 weeks for 30–40% of ulcers and up to 52 weeks for approximately 25% of all DFU [2,77]. Even when healing has occurred, these patients are at high risk for recurrence. It is estimated that between 25 and 42%, 44 and 58%, and 50 and 65% of patients will develop a second ulcer after 1 year, 3 years, and 5 years, respectively [2,6], with the recurrent ulcer being in the same location or on the contralateral foot [2,78].

### 5.1. Microbiology of DFUs and Management

**Figure 4:**
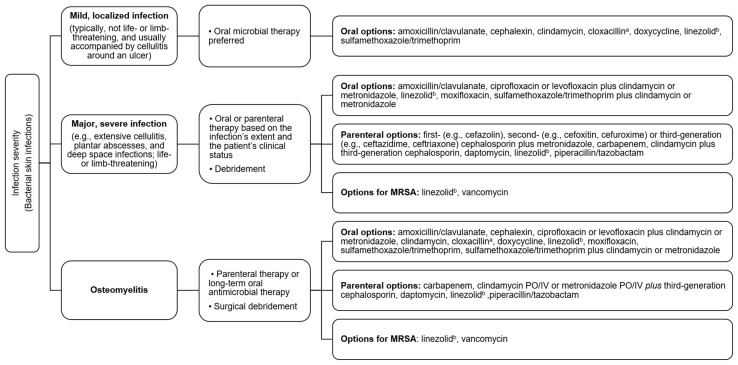
Empiric therapy for bacterial diabetic foot infections based on infection severity. The recommendations may vary based on local prescribing guidelines. IV: intravenous, PO: by mouth, MRSA: Methicillin-resistant *Staphylococcus aureus*. ^a^ Rarely used due to the four-times-a-day dosing schedule, poor bioavailability, and the requirement to be taken on an empty stomach. ^b^ If linezolid is used for more than 2 weeks, myelosuppression (e.g., anemia, thrombocytopenia) should be monitored.

Pathogens that are recovered from a DFI or DFU may be either contaminants (non-replicating) or replicating microbes that have colonized the wound. Microbial testing of the pathogens present within a DFI or a DFU with signs of active infection is needed to identify not only the pathogens present but also to help determine an appropriate systemic antibiotic therapy with antimicrobial sensitivity testing [20,29]. Particularly, testing needs to determine if multidrug-resistant organisms (MDRO), such as methicillin-resistant *Staphylococcus aureus* (MRSA), are present, which can be difficult to treat [20]. Figure 4 summarizes empiric therapy for bacterial diabetic foot infections based on infection severity [79]. However, the antibiotic recommendations (Figure 4) may vary based on local or national prescribing guidelines. Physicians need to practice good antifungal and antibiotic stewardship, keeping with local resistance patterns, to prevent the development of these difficult-to-treat, resistant infections [17,20,29].

### 5.2. Treatments for Superficial Fungal Infections

Both toenail onychomycosis and tinea pedis can be challenging to treat, particularly in diabetic patients. The treatments used to treat non-diabetic patients are also used within the diabetic population, with additional considerations. These patients may have poor peripheral circulation [19], which can decrease the efficacy of systemic therapies, while topical medications may be a viable option for some patients [53]. They may have difficulty accessing their feet due to limited mobility and obesity, potentially limiting the use of topical therapies and preventative self-footcare interventions [80]. Additionally, there are a limited number of studies that have specifically studied onychomycosis treatments in diabetic patients. We summarize current treatment options for both toenail onychomycosis (Table 1) and tinea pedis (Table 2) and treatment considerations for diabetic patients.

#### 5.2.1. Toenail Onychomycosis

Mild-to-moderate toenail onychomycosis, particularly distal and lateral subungual onychomycosis (DLSO), can be managed using oral and topical medications. Standard oral medications include terbinafine, itraconazole, and fluconazole. Terbinafine is the most effective of these three medications [53,81,82]. Topical medications in the USA include ciclopirox nail lacquer, efinaconazole 10% solution, and tavaborole 5% solution [53,81,83].

Oral terbinafine is the first-line treatment for dermatophyte onychomycosis [84]. Itraconazole serves as an alternative therapy and can be used as either continuous or pulse therapy [85]. Efinaconazole 10% topical solution is useful for patients who cannot tolerate oral treatments or have mild to moderate infections [86]. Ciclopirox nail lacquer is less effective than oral treatments but can be beneficial for mild cases or in combination therapy [87]. Tavaborole 5% topical solution is also an option for mild-to-moderate dermatophyte onychomycosis [88,89].

For non-dermatophyte onychomycosis, itraconazole is preferred over terbinafine. Fluconazole is an alternative treatment, especially when non-dermatophyte yeasts like *Candida* species are involved [90]. Topical therapies such as efinaconazole or ciclopirox may be used for mild infections or in combination with oral antifungals [89].

#### 5.2.2. Tinea Pedis

Tinea pedis may precede or accompany onychomycosis [14,59]. The authors recommend following the same protocol as in non-diabetics; however, in some cases, the disease may need to be treated for a longer duration (AKG, personal experience). Also, healthcare providers need to be vigilant about the recurrence of tinea pedis more frequently than in the normal population [14].

### 5.3. Treatment Considerations for Diabetic Patients When Using Oral Antifungals

Many oral medications used for diabetes treatments are metabolized through cytochrome P450 enzymes, which have the potential for interaction with itraconazole and fluconazole. Both itraconazole and fluconazole are metabolized through cytochrome P450 pathways, particularly CYP 3A4 and CYP 2C9, which could lead to interactions with many sulphonylureas as well as some meglitinides and thiazolidinediones [91,92]. For a full list of drug interactions, the reader is referred to specialized texts [84,85,86,87,88,90].

Diabetics may have other co-morbidities, such as renal or liver dysfunction; this may require dosage adjustments, and we direct the reader to specialized texts [84,85,86,87,88,90].

## 6. Role of the Dermatologist in Diabetes Management

As diabetic patients present with compromised vascular, neurological, and immune function, they face not only a higher risk of contracting onychomycosis and tinea pedis but also a significantly higher risk of complications from these infections [14,53,58]. As fungal infections can be progressive, effective treatment with an intent for clinically meaningful improvement is essential for preventing further complications, including wounds and ulcerations caused by nail and skin changes and secondary infections precipitated from these complications [53,58]. These secondary infections can be caused by a variety of pathogens, commonly *Escherichia coli*, *Proteus* spp., and *Staphylococcus aureus*, which is of significant concern when methicillin-resistant strains (MRSA) are present [17,20,29]. Many DFUs with associated infections will be polymicrobial, further complicating treatment [17,20].

Dedicated dermatologist care for diabetic patients to address these infections early will lead not only to a reduction in patient morbidity and mortality but will also lead to a reduction in health costs associated with treating complications that arise. Healthcare costs and access are of concern to disadvantaged populations, as these groups tend to have reduced financial resources and access to insurance.

Regular screening of the patient’s feet for early signs of these infections will also improve outcomes for underserved (disadvantaged) communities and populations. As these populations have reduced access to healthcare, partially due to lower rates of insurance coverage, allowing these infections to progress will lead to higher morbidity and mortality for these patients [7]. Improving access by including the dermatologist as part of the multidisciplinary care team for these patients has the potential to lessen the disparities that exist in healthcare outcomes related to onychomycosis, tinea pedis, and their associated complications. Regular access to care through multidisciplinary care teams has been shown to improve patient health in disadvantaged populations [6,7,9,71].

## 7. Discussion/Closing Remarks

Lower extremity risk assessment in a diabetic for the risk of DFU by an experienced healthcare professional (e.g., dermatologist, family physician, podiatrist) can help identify at-risk patients and help determine best management options, including referral to a specialist multidisciplinary team as appropriate. Early recognition of diabetic foot ulcers reduces morbidity, the risk of hospitalization and amputation, significant disability, and suffering. The patients’ compromised health increases the likelihood of infection [93] and complications [58].

Toenail onychomycosis and tinea pedis have a significant impact on the diabetic patient’s quality of life, not only from a cosmetic perspective, but also from a physical standpoint, as these infections have the potential to cause complications. The prevalence of these infections is higher in diabetic patients than in the general population [63,94,95] and the presence of onychomycosis has been linked to DFU development [61,96]. Early detection and management of risk factors has the potential to improve patient outcomes and reduce the burden on healthcare systems, while also lessening the socio-economic and geographical disparities that currently exist.

## Figures and Tables

**Table 1:** Treatment options for toenail onychomycosis in patients with diabetes.

Topical Options	Oral Options
Efinaconazole 10% solution	Terbinafine 250 mg/day × 12–16 weeks
Tavaborole 5% solution	Itraconazole 200 mg/day or 400 mg/day for 1 week a month, 3–4 pulses; continuous 250 mg/day × 12–16 weeks
Ciclopirox 8% solution, nail lacquer	Fluconazole 150–300 mg/week until the nail plate has grown out, typically 9–12 months
Amorolfine 5% nail lacquer Nail debridement ^a^ Keratolytic agent ^a^ (e.g., salicylic acid)	

^a^ Due to peripheral neuropathy and poor circulation in diabetic patients, nail debridement and the use of keratolytic agents should be performed carefully to avoid injury, wound creation, and subsequent infections.

**Table 2:** Treatment options for tinea pedis in patients with diabetes.

Topical Options	Oral Options
Luliconazole 1% cream	Terbinafine 250 mg/day × 4–6 weeks
Sertaconazole 2% cream	Itraconazole 200 mg/day or 400 mg/day for 1 week a month; continuous 250mg/day 4–6 weeks
Oxiconazole 1% cream	Fluconazole 150–300 mg/week × 4–6 weeks
Naftifine 1% or 2% cream	
Clotrimazole 1% cream or solution	
Miconazole 2% cream	
Terbinafine1% cream, solution, gel, or film-forming solution	
Ciclopirox 1% cream	
Ketoconazole 2% cream	

## Data Availability

No new data were created or analyzed in this study.

## Abbreviations

DM	Diabetes Mellitus
PAD	Peripheral Arterial Disease
DPN	Diabetic Peripheral Neuropathy
DFU	Diabetic Foot Ulcer
DFI	Diabetic Foot Infection
DFO	Diabetic Foot Osteomyelitis
ROS	Reactive Oxygen Species
AGE	Advanced Glycation Endproducts
ANS	Autonomic Nervous System
SINBAD	Site, Ischemia, Neuropathy, Bacterial Infection, and Depth
PEDIS	Perfusion (ischemia), Extent (area), Depth, Infection, Sensation (neuropathy)
MDRO	Multidrug Resistant Organisms
MRSA	Methicillin-resistant *staphylococcus aureus*
IV	Intravenous
PO	Oral
CYP	Cytochrome P450
ABI	Arterial-Brachial Index
WIFI	Wound, Ischemia, Foot Infection
